# CRP as an early indicator for anastomotic leakage after esophagectomy for cancer: a single tertiary gastro-esophageal center study

**DOI:** 10.1007/s00423-023-03176-w

**Published:** 2023-11-15

**Authors:** Motonari Ri, Antonios Tzortzakakis, Ira Sotirova, Andrianos Tsekrekos, Fredrik Klevebro, Mats Lindblad, Magnus Nilsson, Ioannis Rouvelas

**Affiliations:** 1 Department of Clinical Science, Intervention and Technology (CLINTEC), Division of Surgery and Oncology, Karolinska Institutet, Hälsovägen 13, 141 57 Huddinge, Stockholm Sweden; 2 Department of Upper Abdominal Diseases, Karolinska University Hospital, Stockholm, Sweden; 3 Department for Clinical Science, Intervention and Technology (CLINTEC), Division of Radiology, Karolinska Institutet, Stockholm, Sweden; 4 Department of Medical Radiation Physics and Nuclear Medicine, Functional Unit of Nuclear Medicine, Karolinska University Hospital, Stockholm, Sweden; 5 Department of Surgery, Umeå University Hospital, Umeå, Sweden

**Keywords:** Esophageal cancer, Esophagectomy, Anastomotic leakage, C-reactive protein

## Abstract

**Purpose:**

To determine the relationship between postoperative C-reactive protein (CRP) as an early indicator of anastomotic leakage (AL) after esophagectomy for esophageal cancer.

**Methods:**

We reviewed patients diagnosed with esophageal or esophagogastric junctional cancer who underwent esophagectomy between 2006 and 2022 at the Karolinska University Hospital, Stockholm, Sweden. Multivariable logistic regression models estimated relative risk for AL by calculating the odds ratio (OR) with a 95% confidence interval (CI). The cut-off values for CRP were based on the maximum Youden’s index using receiver operating characteristic curve analysis.

**Results:**

In total, 612 patients were included, with 464 (75.8%) in the non-AL (N-AL) group and 148 (24.2%) in the AL group. Preoperative body mass index and the proportion of patients with the American Society of Anesthesiologists physical status classification 3 were significantly higher in the AL group than in the N-AL group. The median day of AL occurrence was the postoperative day (POD) 8. Trends in CRP levels from POD 2 to 3 and POD 3 to 4 were significantly higher in the AL than in the N-AL group. An increase in CRP of ≥ 4.65% on POD 2 to 3 was an independent risk factor for AL with the highest OR of 3.67 (95% CI 1.66–8.38, *p* = 0.001) in patients with CRP levels on POD 2 above 211 mg/L.

**Conclusion:**

Early changes in postoperative CRP levels may help to detect AL early following esophageal cancer surgery.

**Supplementary Information:**

The online version contains supplementary material available at 10.1007/s00423-023-03176-w.

## Introduction

Esophageal cancer is the sixth leading cause of cancer-related death worldwide, and its incidence is on the rise, particularly in Western countries [1]. Esophagectomy with lymph node dissection combined with chemotherapy or chemoradiotherapy is currently considered the standard treatment for locally advanced esophageal cancer [2, 3]. However, despite the introduction of minimally invasive esophagectomy (MIE), which has improved short- and long-term outcomes [4–6], esophagectomy remains one of the most invasive procedures among gastrointestinal surgeries. It is associated with a significant operative mortality [7, 8].

Anastomotic leakage (AL) is a common postoperative complication following esophagectomy, regardless of the surgical approach or reconstruction site [4]. Moreover, AL is also associated with increased mortality rates and a negative impact on long-term survival [9–11]. Therefore, it is crucial to manage AL swiftly and appropriately. Early diagnosis of AL is especially important as it can facilitate prompt intervention and potentially reduce the severity of the complication.

C-reactive protein (CRP), an inflammatory biomarker synthesized in the liver, has been widely recognized as a diagnostic indicator of surgical and infectious complications after abdominal surgery [12, 13]. In the context of esophageal cancer surgery, CRP has been reported as a useful negative indicator for ruling out AL after esophagectomy [14–16]. However, few studies have investigated whether CRP can be a valuable predictor of AL after esophagectomy, particularly in the very early postoperative period [17, 18]. Thus, the potential of CRP as an indicator of AL in this critical period remains to be determined.

This study aims to assess the potential of CRP as an early indicator of complications, specifically AL, following esophagectomy for cancer. The findings of this study have the potential to aid in the early diagnosis and management of AL, ultimately leading to improved patient outcomes in routine clinical practice.

## Methods

### Patients

Between January 2006 and December 2022, all patients who underwent esophagectomy at the Karolinska University Hospital, Sweden, a tertiary gastro-esophageal center, were reviewed in the present study. Inclusion criteria were as follows: patients diagnosed with esophageal or esophagogastric junctional cancer (Siewert type I and II), undergoing a primary esophageal resection and reconstruction with a gastric tube, i.e., patients undergoing thoracoabdominal esophagectomy according to McKeown, or according to Ivor Lewis, or trans-hiatal esophagectomy. Ethical approval was obtained from Stockholm’s Regional Research Ethics Committee (EPN) with reference numbers 2018/970–31/1 and 2022–02634-02.

### Surgical and perioperative management

All surgeries were performed using open, hybrid, or MIE approaches. MIE also included cases with a robot approach. In MIE cases, the Ivor Lewis procedure typically involved a mechanical overlapping side-to-side anastomosis employing a linear stapler, while the McKeown procedure or transhiatal esophagectomy employed either mechanical anastomosis with a linear stapler or hand-sewn anastomosis, depending on the height of the anastomosis and the surgeon’s choice. As part of perioperative management, an enhanced recovery protocol was followed starting from April 2014. Postoperative routine follow-up included daily blood tests and oral contrast swallow imaging on postoperative day 3. In cases where patients exhibited symptoms such as elevated fever, tachycardia, and discharge of saliva or air from the cervical wound, further examinations, including computed tomography (CT) and upper gastrointestinal endoscopy, were performed.

### Definition of anastomotic leakage

AL was defined as a full-thickness gastrointestinal defect involving the esophagus, anastomosis, staple line, or conduit based on the Esophagectomy Complications Consensus Group (ECCG) system [19]. All ALs were included in the study regardless of severity grade and clinical relevance. AL was diagnosed primarily by CT with the application of a small amount of water-soluble oral contrast agent or was identified by the discharge of saliva or gastrointestinal contents from the opened neck wound. Upper gastrointestinal endoscopy was also performed as needed to confirm whether an AL was present.

### Data collection

Patient data, including characteristics, surgical outcomes, and postoperative findings, was collected from information contained in the hospital’s surgical planning system (ORBIT) and the patient chart system (take–care). The clinical and pathological T and N category was assigned according to the TNM classification, the eighth edition established by the Union for International Cancer Control [20]. Postoperative complications with severity grade 2 or more according to the Clavien–Dindo classification (C-D) were regarded as events, while severe complications were defined as C-D ≥ 3 [21]. Medical complications included: pneumonia, pulmonary embolism, and cardiovascular complications.

### Statistical analysis

Continuous variables were depicted as the median and interquartile range (IQR), and frequency percentages were calculated for categorical variables. Continuous variables were compared using the Mann–Whitney *U* or Student’s *t-*test, while the Chi-square test or Fisher’s exact test was conducted for categorical variables. The optimal cut-off values of CRP and changes in CRP were determined based on the maximum Youden’s index using receiver operating characteristic (ROC) curve analysis and calculation of the area under the curve (AUC). Logistic regression models were conducted to estimate the relationship between the exposure variables and AL/all severe complications, yielding the odds ratio (OR) between groups and 95% confidence intervals (CI). Multivariable adjustments were made for the following predefined clinically relevant variables with categorization in all the models mentioned above; age (continuous), sex, body mass index (BMI) (continuous), the American Society of Anesthesiologists physical status classification (ASA-PS), clinical T stage, clinical N stage, neoadjuvant therapy (none, neoadjuvant chemotherapy or neoadjuvant chemoradiotherapy), surgical approach (open, hybrid MIE, or MIE), and the type of surgery (McKeown, Ivor Lewis or trans-hiatal esophagectomy). A *p-*value of 0.05 was considered to indicate a statistically significant difference. All statistical analyses were done using JMP Pro 17 (SAS Institute Japan Ltd, Japan) for Windows.

## Results

### Patient characteristics

Patients’ characteristics are shown in Table 1. In total, 612 patients with 464 in the non-AL (N-AL) group and 148 in the AL group, were included in the study. Patients who underwent MIE totaled 393 cases, including a subset of 57 cases with a totally robotic approach. The AL group had a significantly higher preoperative BMI than the N-AL group. Additionally, a higher proportion of patients with ASA-PS 3 or without neoadjuvant treatment were observed in the AL group compared to the N-AL group.

**Table 1:** Patient characteristics

	N-AL group	AL group	*P* value
	(*n* = 464)	(*n* = 148)	
Age, years [IQR]	67 [60–73]	68.5 [62–73]	0.193
Sex, *n* (%)			0.072
Male	360 (77.6)	125 (84.5)	
Female	104 (22.4)	23 (15.5)	
BMI, kg/m^2^ [IQR]	25.3 [22.6–28.0]	26.1 [23.3–28.6]	**0.047**
ASA-PS, *n* (%)			**0.007**
1	117 (25.2)	29 (19.6)	
2	241 (51.9)	66 (44.6)	
3	106 (22.8)	53 (35.8)	
Clinical T category, *n* (%)			0.346
T0/1	31 (6.7)	16 (10.8)	
T2	59 (12.7)	17 (11.5)	
T3	289 (62.3)	92 (62.2)	
T4	84 (18.1)	22 (14.8)	
Missing	1 (0.2)	1 (0.7)	
Clinical N category, *n* (%)			0.389
N0	191 (41.2)	67 (45.3)	
N +	272 (58.6)	81 (54.7)	
Missing	1 (0.2)	0 (0)	
Clinical M category, *n* (%)			0.438
M0	445 (95.9)	144 (97.3)	
M1	19 (4.1)	4 (2.7)	
Neoadjuvant therapy, *n* (%)			**0.027**
None	120 (25.9)	55 (37.2)	
Chemotherapy	147 (31.7)	37 (25)	
Chemoradiotherapy	197 (42.4)	56 (37.8)	
Preoperative CRP, mg/L [IQR]	2.5 [1–7]	3 [1–7]	0.803

*N-AL* non-anastomotic leakage, *AL* anastomotic leakage, *IQR* interquartile range, *BMI* body mass index, *ASA-PS* the American Society of Anesthesiologists physical status classification, *CRP* C-reactive protein

Bold numbers indicate statistical significance

### Surgical outcomes and pathological findings

Table 2 presents information on the surgical and pathological outcomes. The median day of AL occurrence was the postoperative day (POD) 8 with an IQR of 6 to 10 days. CRP levels on POD 2, 3, and 4 were significantly higher in the AL than in the N-AL group (*p* < 0.001). Patients in the AL group had a greater change in CRP levels from POD 2 to 3 and POD 3 to 4 compared to those in the N-AL group (POD 2 to 3 *p* < 0.001, POD 3 to 4 *p* = 0.026). The AL group had significantly higher 30-day (5.4% vs 1.9%, *p* = 0.026) and 90-day (10.8% vs 5.2%, *p* = 0.016) mortality rates than the N-AL group. Postoperative hospital stay was significantly longer in the AL than in the N-AL group (median 29 vs 13 days; *p* < 0.001).

**Table 2:** Operative outcomes and pathological findings

	N-AL group	AL group	*P* value
	(*n* = 464)	(*n* = 148)	
Surgical approach, *n* (%)			0.210
Open	138 (29.7)	33 (22.3)	
Hybrid MIE	35 (7.5)	13 (8.8)	
MIE	291 (62.7)	102 (68.9)	
Type of esophagectomy, *n* (%)			0.234
McKeown	128 (27.6)	51 (34.5)	
Ivor Lewis	292 (62.9)	82 (55.4)	
Transhiatal	44 (9.5)	15 (10.1)	
Conversion to open technique, *n* (%)	14 (3.0)	3 (2.0)	0.523
Operative duration, min [IQR]	420 [360–495]	431 [385–512]	0.121
Intraoperative blood loss, ml [IQR]	200 [100–500]	195 [75–400]	0.105
CRP on POD 2^*^, mg/L [IQR]	153 [110–204]	178 [130–239]	** < 0.001**
CRP on POD 3^*^, mg/L [IQR]	160 [113–219]	209 [147–276]	** < 0.001**
CRP on POD 4^*^, mg/L [IQR]	142 [94–194]	197 [132–255]	** < 0.001**
Trend in CRP between POD 2 and 3, % [IQR]	3.1 [− 13.5 to 21.9]	10.7 [− 2.9 to 31.5]	** < 0.001**
Trend in CRP between POD 3 and 4, % [IQR]	− 13.1 [− 26.3 to 3.4]	− 8.8 [− 21.0 to 6.3]	**0.026**
Medical complications^†^ (C-D ≥ 2), *n* (%)	131 (28.2)	41 (27.7)	0.901
Date of leakage diagnosis, postoperative day	-	8 [6–10]	-
Postoperative hospital stay, days	13 [10–21.8]	29 [18–48]	** < 0.001**
30-day mortality, *n* (%)	9 (1.9)	8 (5.4)	**0.026**
90-day mortality, *n* (%)	24 (5.2)	16 (10.8)	**0.016**
Histological tumor type			0.609
Adenocarcinoma	369 (79.5)	112 (75.6)	
Squamous cell carcinoma	90 (19.4)	34 (23.0)	
Others	5 (1.1)	2 (1.4)	
Pathological T category, *n* (%)			0.238
T0	74 (16.1)	26 (17.8)	
T1	63 (13.7)	27 (18.5)	
T2	66 (14.4)	27 (18.5)	
T3	217 (47.3)	57 (39.0)	
T4	39 (8.5)	9 (6.2)	
Pathological N category, *n* (%)			0.309
N0	220 (47.9)	82 (56.2)	
N1	89 (19.4)	27 (18.5)	
N2	68 (14.8)	18 (12.3)	
N3	82 (17.9)	19 (13.0)	
Pathological stage, *n* (%)			0.152
Stage 0	62 (13.5)	21 (14.4)	
Stage I	45 (9.8)	24 (16.4)	
Stage II	126 (27.5)	42 (28.8)	
Stage III	130 (28.3)	37 (25.3)	
Stage IV	96 (20.9)	22 (15.1)	

*N-AL* non-anastomotic leakage, *AL* anastomotic leakage, *MIE* minimally invasive esophagectomy, *IQR* interquartile range, *CRP* C-reactive protein, *POD* postoperative day, *C-D* Clavien–Dindo classification

^*^In CRP levels on POD 2, 3, and 4, there were 2, 6, and 15 patients, respectively, with missing data

^†^Medical complications were defined as pneumonia, pulmonary embolism, and cardiovascular complications

Bold numbers indicate statistical significance

### Predictors for anastomotic leakage and severe complications

Tables 3 and 4 show the logistic regression analysis for AL and severe complications (C-D ≥ 3). The calculations of optimal cutoff values for the exposure variables are shown in Supplementary Figs. 1 and 2. The analysis of the trend in CRP on POD 2 to 3 and the trend in CRP on POD 3 to 4 was limited to patients whose CRP levels were higher than the optimal cutoff value on POD 2 and POD 3, respectively. The sensitivity, specificity, positive predictive value, and negative predictive value of CRP for diagnosing AL and severe complications are shown in Supplementary Tables 1 and 2. The multivariable analysis identified CRP levels of ≥ 211 mg/L for POD 2, ≥ 222 mg/L for POD 3, ≥ 190 mg/L for POD 4, and trends in the CRP of ≥ 4.65% on POD 2 to 3 as independent risk factors for AL (Table 3). Trends in the CRP of ≥ 4.65% on POD 2 to 3 had the highest OR of 3.67 (95% CI 1.66–8.38, *p* = 0.001), which was also the highest among all other variables (Supplementary Table 3). Accordingly, CRP ≥ 161 mg/L for POD 2, CRP ≥ 208 mg/L for POD 3, CRP ≥ 189 mg/L for POD 4, trends in CRP ≥ 2.85% on POD 2 to 3, and trends in CRP ≥  − 13.7% on POD 3 to 4 were all independent risk factors for severe complications (Table 4). Among those, the OR for CRP ≥ 189 mg/L on POD 4 had the highest value, 3.40 (95% CI 2.36–4.94, *p* < 0.001).

**Table 3:** Risk of anastomotic leakage

	CRP on POD 2			CRP on POD 3			CRP on POD 4			Trend in CRP on POD 2 to 3*			Trend in CRP on POD 3 to 4^†^		
	< 211 mg/L	≥ 211 mg/L	*P*-value	< 222 mg/L	≥ 222 mg/L	*P*-value	< 190 mg/L	≥ 190 mg/L	*P*-value	< 4.65%	≥ 4.65%	*P*-value	< − 25.7%	≥ − 25.7%	*P*-value
	OR	OR		OR	OR		OR	OR		OR	OR		OR	OR	
	(95% CI)	(95% CI)		(95% CI)	(95% CI)		(95% CI)	(95% CI)		(95% CI)	(95% CI)		(95% CI)	(95% CI)	
Crude	1.00	2.21	** < 0.001**	1.00	2.84	** < 0.001**	1.00	3.49	** < 0.001**	1.00	4.59	** < 0.001**	1.00	2.07	0.095
	(Reference)	(1.49–3.29)		(Reference)	(1.93–4.18)		(Reference)	(2.37–5.15)		(Reference)	(2.29–9.19)		(Reference)	(0.87–4.94)	
Adjusted	1.00	2.17	** < 0.001**	1.00	2.65	** < 0.001**	1.00	3.30	** < 0.001**	1.00	3.67	**0.001**	1.00	2.09	0.119
	(Reference)	(1.42–3.31)		(Reference)	(1.77–3.99)		(Reference)	(2.21–4.95)		(Reference)	(1.66–8.38)		(Reference)	(0.83–5.74)	

Multivariable logistic regression models were adjusted for age, sex, body mass index, ASA score, clinical T and N status, neoadjuvant therapy, surgical approach, and type of surgery

*CRP* C-reactive protein, *POD* postoperative day, *OR* odds ratio, *CI* confidence interval

^*^Only patients above the cutoff value of CRP 211 mg/L on POD 2 were included

^†^Only patients above the cutoff value of CRP 222 mg/L on POD 3 were included

Bold numbers indicate statistical significance

**Table 4:** Risk of all severe complications (C-D ≥ 3)

	CRP on POD 2			CRP on POD 3			CRP on POD 4			Trend in CRP on POD 2 to 3*			Trend in CRP on POD 3 to 4^†^		
	< 161 mg/L	≥ 161 mg/L	*P*-value	< 208 mg/L	≥ 208 mg/L	*P*-value	< 189 mg/L	≥ 189 mg/L	*P*-value	< 2.85%	≥ 2.85%	*P*-value	< − 13.7%	≥ − 13.7%	*P*-value
	OR	OR		OR	OR		OR	OR		OR	OR		OR	OR	
	(95% CI)	(95% CI)		(95% CI)	(95% CI)		(95% CI)	(95% CI)		(95% CI)	(95% CI)		(95% CI)	(95% CI)	
Crude	1.00	2.05	** < 0.001**	1.00	3.01	** < 0.001**	1.00	3.37	** < 0.001**	1.00	2.25	** < 0.001**	1.00	2.23	**0.006**
	(Reference)	(1.48–2.84)		(Reference)	(2.13–4.26)		(Reference)	(2.36–4.79)		(Reference)	(1.40–3.61)		(Reference)	(1.26–3.95)	
Adjusted	1.00	1.58	** < 0.001**	1.00	2.98	** < 0.001**	1.00	3.40	** < 0.001**	1.00	2.30	**0.002**	1.00	2.54	**0.004**
	(Reference)	(1.32–2.61)		(Reference)	(2.08–4.34)		(Reference)	(2.36–4.94)		(Reference)	(1.37–3.89)		(Reference)	(1.35–4.87)	

Multivariable logistic regression models were adjusted for age, sex, body mass index, ASA score, clinical T and N status, neoadjuvant therapy, surgical approach, and type of surgery

*C-D* Clavien–Dindo classification, *CRP* C-reactive protein, *POD* postoperative day, *OR* odds ratio, *CI* confidence interval

^*^Only patients above the cutoff value of CRP 161 mg/L on POD 2 were included

^†^Only patients above the cutoff value of CRP 208 mg/L on POD 3 were included

Bold numbers indicate statistical significance

## Discussion

The present study examined the association between postoperative CRP levels and AL in patients who underwent surgery for esophageal cancer. The findings indicate that changes in CRP during the very early postoperative period, specifically between postoperative days 2 to 3, may be the most reliable positive indicator of AL following esophagectomy for cancer.

The usefulness of postoperative CRP levels as a negative predictor of AL after esophagectomy has been reported in previous studies [14–16]. Aiolfi et al. [14], in a Bayesian meta-analysis, indicated that CRP < 176 mg/L on POD 3 and CRP < 132 mg/L on POD 5 might be useful for ruling out AL after esophagectomy. Moreover, Rat et al. [16] suggested that CRP < 130 mg/L on POD 5 is a useful negative predictor for AL with a negative predictive value of 96% in 585 patients who underwent Ivor Lewis esophagectomy. Conversely, studies regarding CRP as a positive predictor for AL are limited. Prochazka et al. [17] reported that in 40 patients undergoing MIE McKeown esophagectomy, CRP on POD 5 was significantly higher in the AL group than in the N-AL group. In comparison, only one patient presented clinical symptoms at the same time among all 11 patients with AL [17]. Furthermore, Park et al. [18] demonstrated that CRP levels around 170 mg/L on POD 3 may be a predicting factor for AL after esophagectomy. However, it should be noted that the former study was limited to univariate analysis, and the latter had a very small sample size of 46 cases, which makes the usefulness of CRP as an indicator for detecting AL uncertain.

**Fig. 1:**
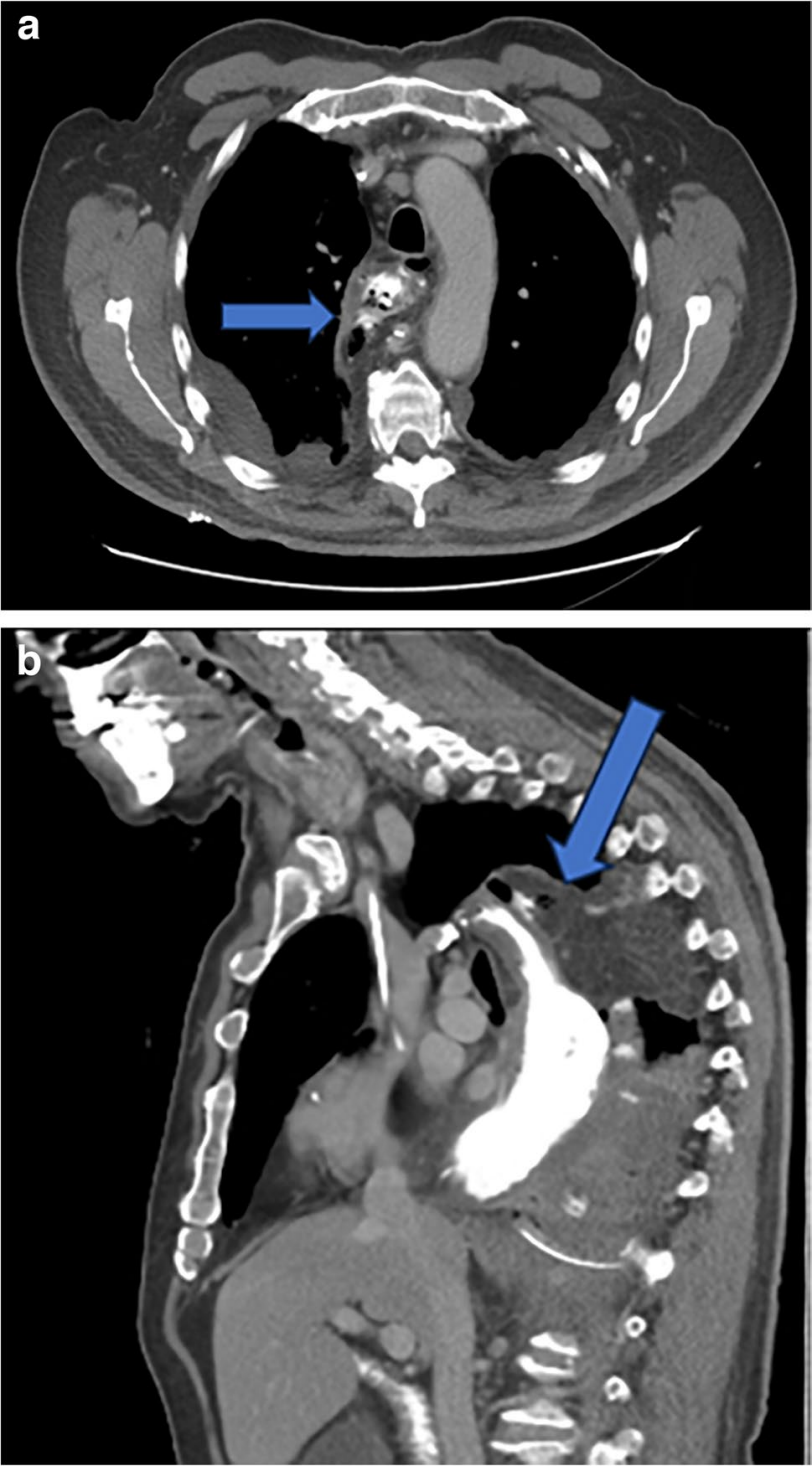
Computed tomography after esophagectomy showing anastomotic leakage. A 77-year-old man operated with minimally invasive esophagectomy using the Ivor Lewis technique. Postoperative day 5, anastomotic leakage (arrows) was depicted in computed tomography with oral contrast, axial (**a**), and sagittal (**b**) reformations

AL is one of the potentially fatal complications following esophageal cancer surgery. When AL occurs, the contents of the gastrointestinal tract can escape into the deep compartments, leading to serious infections such as empyema and mediastinitis (Fig. 1). These infections can sometimes trigger sepsis, resulting in severe conditions such as multiple organ failure and acute respiratory distress syndrome, ultimately leading to death. The 90-day postoperative mortality rate for patients who develop AL after esophageal cancer surgery is not low and has been reported to be 5.3 to 11.7%, which is in line with the result of this study [22, 23]. Early detection of AL and timely implementation of appropriate interventions are demanded to avert the potentially lethal course of AL.

The median time to AL diagnosis was on POD 8 in the present study, a similar result to previous reports by Noble et al. and Tsujimoto et al. [22, 23] indicating that AL diagnosis occurs on POD 7. Considering the outcome of this study, early changes in CRP may be an early indicator of AL. Alternatively, there is a possibility that the early postoperative inflammatory state may be involved in the development of anastomotic leakage and CRP would thus be a prognosticator. High CRP levels on POD 6 and POD 7 were, as expected, also independent risk factors for AL with significant odds ratios (data not shown) since the leakage at this time of the postoperative course is established. However, the current study aimed to identify AL as early as possible and intervene promptly before the patient develops sepsis, aiming for favorable outcomes. The current study suggests that already on POD 3 CRP trends may reveal AL and consequently severe complications. Therefore, it is crucial to maintain a high level of suspicion and to monitor CRP levels closely for the early and timely detection of AL.

The diagnosis of AL is generally confirmed through other modalities of examination, such as oral contrast swallow, followed by CT and endoscopy [24]. However, there is no standardized approach to diagnosing AL, and sometimes asymptomatic cases of AL may also exist [25]. Based on the trend criteria of CRP obtained from this study, a lower threshold may be allowed to perform these confirming examinations earlier to diagnose AL even without symptoms. This can enable early intervention benefiting the patient. Additionally, if a definitive diagnosis of AL cannot be confirmed through these additional modalities, more intense surveillance can be implemented with repeated radiology and endoscopy to prevent unnecessary delay in diagnosis and subsequent deterioration of the patient’s condition.

Although the basic treatment strategy for AL is based on the closure or coverage of the anastomotic defect and the drainage of leaked fluids outside the gastrointestinal tract, the management strategy for AL can vary widely, especially depending on the location of the anastomosis, the size of AL, time from the onset of AL, the severity of symptoms and the presence of conduit ischemia [25]. As endoscopic treatments, self-expanding metal stents and endoscopic vacuum therapy (EVT) using a sponge are available options [26, 27]. In our previous case series, we reported the improvement of CRP through EVT [28]. It is known that AL following esophageal cancer surgery can have a negative impact on long-term outcomes due to prolonged inflammation [10, 29, 30]. Therefore, early intervention for AL provides an increased opportunity for effective management and potentially improves long-term outcomes.

There are several limitations to this study. First, being a retrospective study from a single institution, it cannot eliminate the possibility of selection bias and residual confounding. Second, although this study had a larger sample size compared to previous reports, it is possible that more statistical power was required to determine the accuracy of the AUCs obtained from each CRP cutoff value, especially considering that all AUC values, except for one, were below 0.7. Third, information on other comorbidities was not included in the patients’ preoperative status, which may have influenced the outcome of the present study. The strength of the current study is that it has been conducted in an expert center, and practically the same experienced team of surgeons has performed all the operations. A larger study across multiple centers is warranted to provide more definitive results and address the present study’s limitations.

In conclusion, very early postoperative changes in CRP may be useful in identifying AL following esophageal cancer surgery.

### Supplementary Information

Below is the link to the electronic supplementary material.

Supplementary file1 (DOCX 179 KB)

## Data Availability

Data are available upon request.
