# Supplementary material for: CRP as an early indicator for anastomotic leakage after esophagectomy for cancer: a single tertiary gastro-esophageal center study
Source: Langenbecks Arch Surg. 2023 Nov 15;408(1):436. doi: 10.1007/s00423-023-03176-w (PMC10645624; doi:10.1007/s00423-023-03176-w)
Supplement: Supplementary file 1 — Supplementary file1 (DOCX 179 KB) [file 423_2023_3176_MOESM1_ESM.docx]

**Supplementary figure 1. ROC curves for predicting AL after esophagectomy
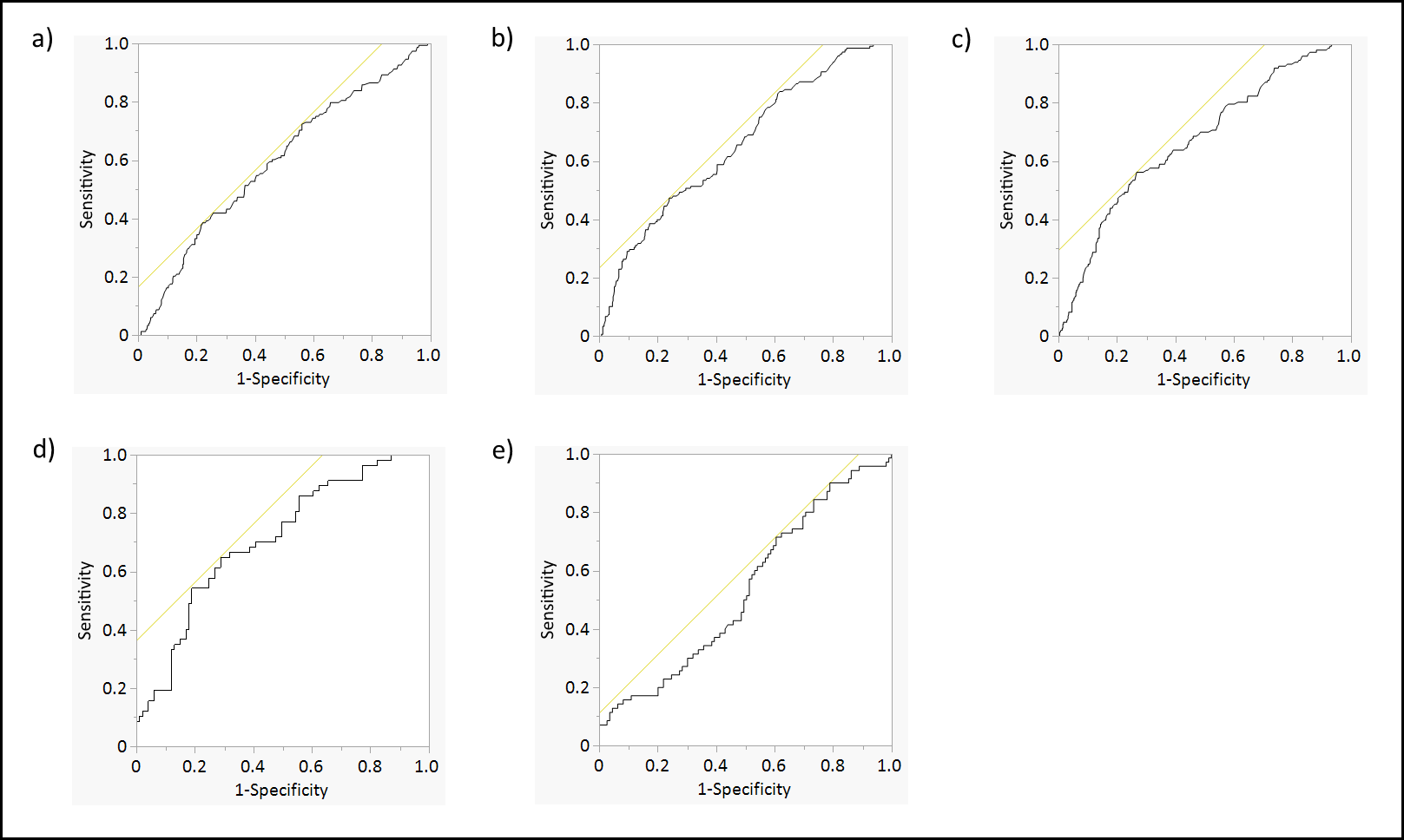
**

a) CRP on POD 2 (AUC=0.594, the cut-off value of CRP; 211 mg/L)

b) CRP on POD 3 (AUC=0.654, the cut-off value of CRP; 222 mg/L)

c) CRP on POD 4 (AUC=0.669, the cut-off value of CRP; 190 mg/L)

d) Trends in CRP 2 to 3 in patients above the cutoff value of CRP 211 mg/L on POD 2 (AUC=0.709, the cut-off value of CRP trend; 4.65 %)

e) Trends in CRP 3 to 4 in patients above the cutoff value of CRP 222 mg/L on POD 3 (AUC=0.532, the cut-off value of CRP trend; -25.7 %)

ROC, receiver operating characteristics; AL, anastomotic leakage; CRP, C-reactive protein; POD, postoperative day; AUC, area under the curve

**Supplementary figure 2. ROC curves for predicting severe complications after esophagectomy**

**
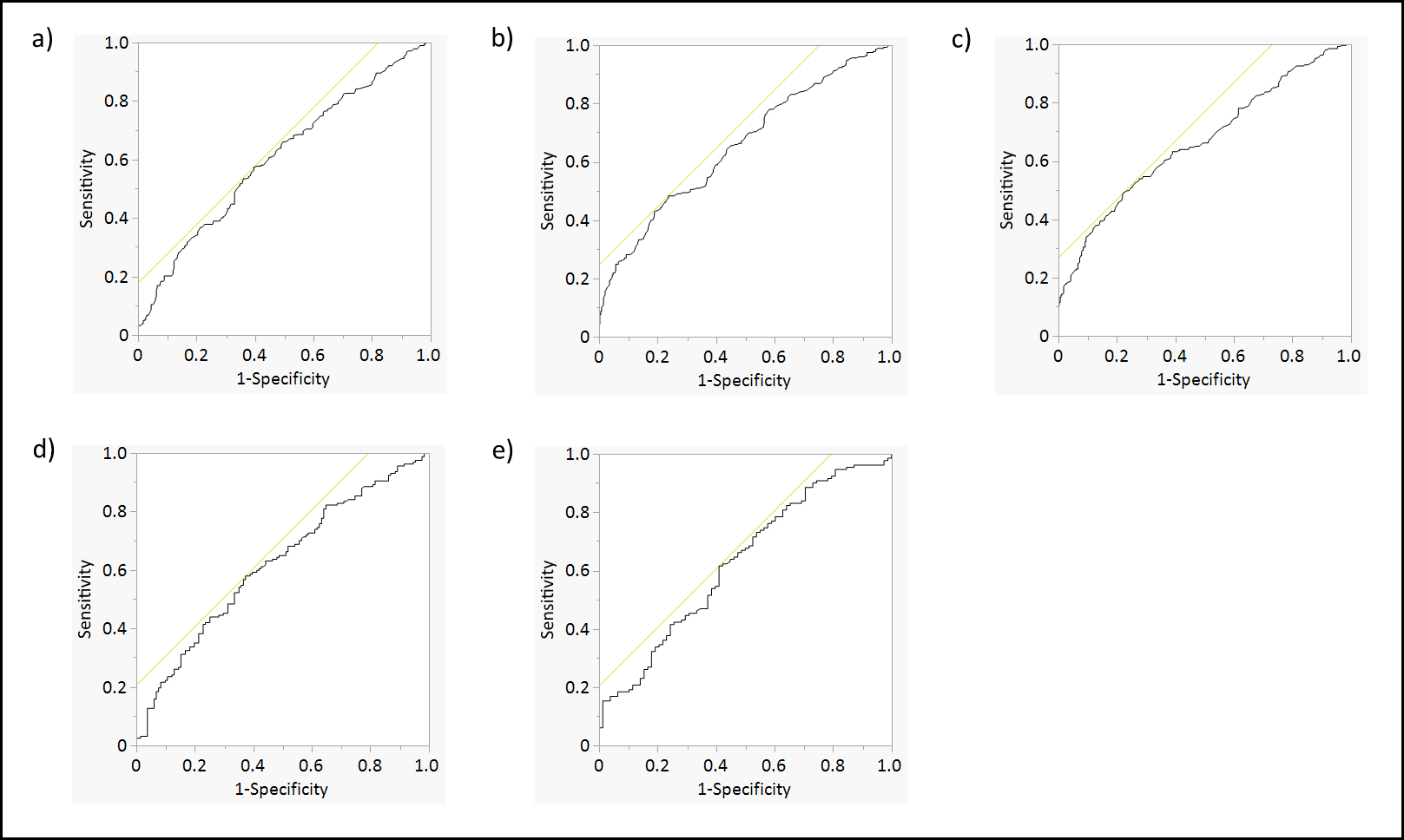
**

a) CRP on POD 2 (AUC=0.607, the cut-off value of CRP; 161 mg/L)

b) CRP on POD 3 (AUC=0.655, the cut-off value of CRP; 208 mg/L)

c) CRP on POD 4 (AUC=0.668, the cut-off value of CRP; 189 mg/L)

d) Trends in CRP 2 to 3 in patients above the cutoff value of CRP 161 mg/L on POD 2 (AUC=0.616, the cut-off value of CRP trend; 2.85 %)

e) Trends in CRP 3 to 4 in patients above the cutoff value of CRP 208 mg/L on POD 3 (AUC=0.636, the cut-off value of CRP trend; -13.7 %)

ROC, receiver operating characteristics; CRP, C-reactive protein; POD, postoperative day; AUC, area under the curve

**Supplementary table 1. Performance of CRP for diagnosing AL**

|  | Sensitivity (%) | Specificity (%) | PPV (%) | NPV (%) |
| --- | --- | --- | --- | --- |
| CRP on POD 2 > 211 mg/L | 38.5 | 77.9 | 35.9 | 79.8 |
| CRP on POD 3 > 222 mg/L | 47.3 | 76.0 | 38.9 | 81.7 |
| CRP on POD 4 > 190 mg/L | 56.2 | 73.2 | 40.4 | 83.8 |
| Trend in CRP on POD 2 to 3 > 4.65 %* | 64.9 | 71.3 | 56.1 | 78.3 |
| Trend in CRP on POD 3 to 4 > -25.7 %^†^ | 88.6 | 21.1 | 41.9 | 74.2 |

*Only patients above the cutoff value of CRP 211 mg/L on POD 2 were included.

†Only patients above the cutoff value of CRP 222 mg/L on POD 3 were included.

CRP, C-reactive protein; AL, anastomotic leakage; PPV, positive predictive value; NPV, negative predictive value; POD, postoperative day

**Supplementary table 2. Performance of CRP for diagnosing severe complications (C-D≥3)**

|  | Sensitivity (%) | Specificity (%) | PPV (%) | NPV (%) |
| --- | --- | --- | --- | --- |
| CRP on POD 2 > 161 mg/L | 57.4 | 60.4 | 54.6 | 63.0 |
| CRP on POD 3 > 208 mg/L | 48.4 | 76.3 | 62.6 | 64.3 |
| CRP on POD 4 > 189 mg/L | 49.1 | 77.7 | 64.4 | 65.1 |
| Trend in CRP on POD 2 to 3 > 2.85 %* | 57.3 | 62.6 | 64.8 | 55.0 |
| Trend in CRP on POD 3 to 4 > -13.7 %^†^ | 60.8 | 59.0 | 71.2 | 47.4 |

*Only patients above the cutoff value of CRP 161 mg/L on POD 2 were included.

†Only patients above the cutoff value of CRP 208 mg/L on POD 3 were included.

CRP, C-reactive protein; C-D, Clavien-Dindo classification; PPV, positive predictive value; NPV, negative predictive value; POD, postoperative day

**Supplementary table 3. Risk of anastomotic leakage in patients above the cutoff value of CRP 211 mg/L on POD 2**

|  | Univariable analysis | | |  | Multivariable analysis | | |
| --- | --- | --- | --- | --- | --- | --- | --- |
|  | OR | 95% CI | *p* value |  | OR | 95% CI | *p* value |
| Age | 0.98 | - | 0.218 |  | 1.01 | 0.96-1.05 | 0.696 |
| Sex |  |  |  |  |  |  |  |
| Male | 1.00 | - | - |  | 1.00 | - | - |
| Female | 0.55 | 0.20-1.47 | 0.229 |  | 0.81 | 0.25-2.40 | 0.706 |
| BMI | 0.95 | - | 0.163 |  | 1.00 | 0.91-1.08 | 0.805 |
| ASA score |  |  |  |  |  |  |  |
| 1 | 1.00 | - | - |  | 1.00 | - | - |
| 2 | 0.84 | 0.35-2.02 | 0.703 |  | 1.00 | 0.35-2.91 | 0.985 |
| 3 | 3.07 | 1.23-7.67 | 0.015 |  | 3.33 | 1.05-11.2 | 0.041 |
| Clinical T factor |  |  |  |  |  |  |  |
| 0-2 | 1.00 | - | - |  | 1.00 | - | - |
| 3 | 1.13 | 0.49-2.60 | 0.782 |  | 1.29 | 0.38-4.60 | 0.680 |
| 4 | 0.71 | 0.23-2.21 | 0.551 |  | 1.10 | 0.23-5.23 | 0.904 |
| Clinical N factor |  |  |  |  |  |  |  |
| 0 | 1.00 | - | - |  | 1.00 | - | - |
| 1 or more | 0.66 | 0.34-1.28 | 0.219 |  | 0.77 | 0.31-1.92 | 0.574 |
| Neoadjuvant treatment |  |  |  |  |  |  |  |
| None | 1.00 | - | - |  | 1.00 | - | - |
| Chemotherapy | 0.44 | 0.18-1.05 | 0.061 |  | 0.48 | 0.15-1.46 | 0.199 |
| Chemoradiotherapy | 0.89 | 0.41-1.91 | 0.762 |  | 0.78 | 0.26-2.28 | 0.647 |
| Surgical approach |  |  |  |  |  |  |  |
| Open | 1.00 | - | - |  | 1.00 | - | - |
| Hybrid MIE | 1.90 | 0.57-6.37 | 0.296 |  | 1.63 | 0.35-7.18 | 0.525 |
| MIE | 1.93 | 0.91-4.11 | 0.085 |  | 1.39 | 0.51-3.81 | 0.519 |
| Type of operation |  |  |  |  |  |  |  |
| McKeown | 1.00 | - | - |  | 1.00 | - | - |
| Ivor Lewis | 1.28 | 0.62-2.64 | 0.506 |  | 1.17 | 0.48-2.84 | 0.735 |
| Transhiatal | 1.30 | 0.45-3.73 | 0.622 |  | 0.70 | 0.15-2.86 | 0.621 |
| Trend in CRP between POD 2-3 |  |  |  |  |  |  |  |
| 4.65% < | 1.00 | - | - |  | 1.00 | - | - |
| 4.65% or more | 4.59 | 2.29-9.19 | <0.001 |  | 3.67 | 1.66-8.38 | **0.001** |

CRP, C-reactive protein; POD, postoperative day; OR, odds ratio; CI, confidence interval; BMI, body mass index; ASA-PS, the American Society of Anesthesiologists physical status classification; MIE, minimally invasive esophagectomy
